# Diversity and life strategies of cyanobacteria and bryophytes within biocrusts in the context of mining tailings disasters in Brazil

**DOI:** 10.1111/plb.70037

**Published:** 2025-05-09

**Authors:** M. F. Oliveira, L. A. Souza, C. C. Figueredo, A. S. Maciel‐Silva

**Affiliations:** ^1^ Programa de Pós‐Graduação em Biologia Vegetal, Instituto de Ciências Biológicas Universidade Federal de Minas Gerais Belo Horizonte Minas Gerais Brazil; ^2^ Instituto Federal de Educação, Ciência e Tecnologia Goiano, Campus Rio Verde Rio Verde Goiás Brazil; ^3^ Departamento de Botânica, Instituto de Ciências Biológicas Universidade Federal de Minas Gerais Belo Horizonte Minas Gerais Brazil

**Keywords:** Biological soil crusts, Fundão Dam, liverworts, mosses, pioneer organisms

## Abstract

- This study investigates the impact of the Fundão Dam rupture in Brazil, one of the most severe industrial disasters globally, focusing on cryptogamic pioneer organisms, which are vital for forming biological soil crusts (biocrusts). These communities, found in superficial soil layers, play a crucial role in carbon and nitrogen fixation, enhancing soil aggregation, retaining moisture, and facilitating vascular plant establishment, making them essential tools for restoration efforts in mining tailings. Within this framework, we compared two sites – one impacted (IS) and one preserved (PS) – focusing on soil chemistry, biocrust taxa, and the life strategies of bryophytes and cyanobacteria.
- Soil was analysed for macro‐ and micronutrients, organic matter, and cation exchange capacity (CEC and ECEC). Biocrusts were identified through microscopy. Bryophyte life strategies were categorized based on traits such as spore size, reproductive strategy, and life form, while cyanobacteria were classified as pioneers, stayers, or stochastic species.
- Our findings revealed diverse biocrust species at both sites, with fugitive and colonist acrocarpous mosses dominating at IS, whereas perennial pleurocarpous mosses and leafy liverworts were exclusive to PS. Cyanobacterial life strategies were similar across sites. The impacted site had lower phosphorus levels and reduced CEC and ECEC compared to the preserved site.
- Four years after the rupture, bryophyte diversity, life strategies, and soil parameters still differ between IS and PS, highlighting the lasting impact of mining tailings. Given the ecological role of biocrusts, their inclusion in monitoring and restoration efforts is crucial for ecosystem recovery.

## Graphical abstract

Biocrust communities show notable distinctions between sites impacted by mining tailings and unimpacted areas in Brazil, encompassing differences not only in diversity but also in terms of ecological life strategies.

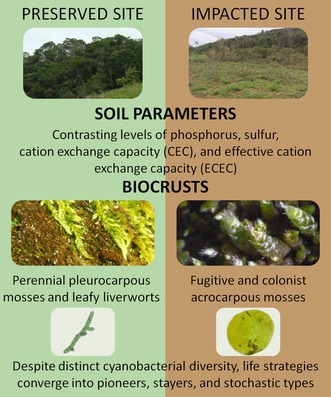

## INTRODUCTION

In November 2015, the rupture of the Fundão Dam in Brazil released 43 million m^3^ of iron ore mining residues (Cruz *et al*. 2020), which travelled over 650 km through the Gualaxo do Norte, Carmo, and Doce rivers before reaching the Atlantic Ocean (Carmo *et al*. 2017). The mining tailings contaminated soils and water, covering and suppressing native vegetation (Cruz *et al*. 2020). With low organic matter, high pH, and elevated levels of ether amines and sodium, the tailings have hindered plant and microbial establishment (Santos *et al*. 2019; Couto *et al*. 2021). Changes in habitat conditions can impact species persistence if alterations exceed their tolerance range (Lynch & Gabriel 1987; Chapin III *et al*. 1993).

Previous studies have highlighted the impact of the Fundão Dam failure on aquatic (e.g., Santos *et al*. 2022) and terrestrial (e.g., Prado *et al*. 2023) ecosystems, with restoration efforts primarily focused on improving tailings conditions in terrestrial environments to restore the native Atlantic Forest (e.g., Scotti *et al*. 2020). Phytoremediation, in particular, has improved sediment fertility, aggregation, and stabilization (Gomes *et al*. 2024). However, pioneer soil organisms, such as those that form biological soil crusts (biocrusts), remain largely unexplored in the context of mining‐impacted areas.

Biocrusts are communities of bryophytes, algae, lichens, cyanobacteria, bacteria, fungi, and archaea interacting with surface soil particles (Weber *et al*. 2022). These organisms are highly resilient in water‐scarce, sun‐exposed ecosystems (Büdel *et al*. 2016; Seppelt *et al*. 2016), with traits that help them survive such harsh conditions (Green & Proctor 2016). For example, mosses and liverworts possess structures that help them endure water scarcity (Vitt *et al*. 2014; Seppelt *et al*. 2016), while cyanobacteria produce exopolysaccharides to protect against solar radiation, temperature changes, and desiccation (Büdel *et al*. 2016; Singh 2018). These traits form consistent life strategies (During 1979; Büdel *et al*. 2016), reflecting species' tolerance ranges (Keddy 1992; Lavorel & Garnier 2002).

In addition to their resilience, biocrusts contribute to ecosystems by fixing nitrogen (Barger *et al*. 2016) and carbon (Sancho *et al*. 2016), aggregating soil (Belnap & Büdel 2016), and retaining moisture (Chamizo *et al*. 2016). As a result, they can play a crucial role in ecosystem restoration (Antoninka *et al*. 2020). Their presence and use in mining‐impacted areas has been studied globally (Liao, Chen, *et al*. 2024), but biocrusts remain poorly documented in Brazilian mining tailings. Only six moss species (Oliveira *et al*. 2024), along with three cyanobacteria and one algal genus (Couto *et al*. 2021), were recorded in the Fundão Dam tailings. These findings are limited, highlighting the need for further research on biocrust dynamics in these tailings.

Within this framework, we aimed to examine the diversity and life strategies of biocrust species at two sites along the Gualaxo do Norte River: one impacted (IS) and one preserved (PS). Our objectives were to: (i) characterize the soil surfaces where biocrusts thrive; (ii) identify bryophyte and cyanobacteria taxa within biocrusts; and (iii) compare their life strategies. We hypothesize that life strategies differ between sites, with pioneer species prevailing at IS and perennial species at PS. This study pioneers biocrust research in Brazilian mining tailings, particularly regarding diversity and life strategies, offering valuable insights into their ecological dynamics and potential for ecosystem restoration.

## MATERIAL AND METHODS

### Studied sites

The study sites experience distinct dry and rainy seasons, with an average temperature of 19.7°C and annual precipitation of 1375 mm (accessed through WorldClim 2). Specifically, the sites are located in the Paracatu de Baixo district, Mariana, representing both unimpacted and mining tailings‐impacted fragments of Atlantic Forest near the Gualaxo do Norte River. Both sites have a long history of livestock grazing. The preserved site (Fig. S1A,B; 20°18′47″ S, 43°12′02″ W), free from tailings, is a forest edge fragment, while the impacted site (Fig. S1C,D; 20°18′22″ S, 43°13′22″ W), contaminated by tailings, is situated near the Gualaxo do Norte River.

### Soil chemical characterization

In March 2020, topsoil samples (~300 g each) were collected beneath biocrusts from each study site, with a total of five samples per site. Samples, spaced at least 2 m apart, were taken from the 0–20 cm layer using a gardening shovel and a PVC pipe (15 cm diameter, 10 cm height). After sieving through a 2 mm mesh, the soil was stored in labelled paper bags, dried at 40°C for 72 h, and sent to the soil analysis laboratory at Universidade Federal de Lavras, Brazil, for chemical analyses following Claessen *et al*. (1997) and Santos *et al*. (2018).

Soil pH was measured in suspensions with water, KCl, and CaCl_2_ at a 1:2.5 soil‐to‐solution ratio. Phosphorus (P), sodium (Na), potassium (K), iron (Fe), zinc (Zn), manganese (Mn), and copper (Cu) were extracted with Mehlich‐1 solution, while calcium (Ca), magnesium (Mg), and aluminium (Al) used a 1 mol L^−1^ KCl solution. Sulfur (S) was extracted with monocalcium phosphate in acetic acid, and boron (B) with hot water. Total acidity (H + Al) was determined by the SMP buffer method. The sum of exchangeable bases (SB) comprised Ca^2+^, Mg^2+^, and K^+^, while effective cation exchange capacity (ECEC) included SB and Al^3+^. Cation exchange capacity at pH 7.0 (CEC) was obtained with ammonium acetate at pH 7.0. Base saturation (BSI) and aluminium saturation (ASI) were calculated as SB and Al^3+^ proportions relative to CEC. Organic matter (OM) was quantified by wet oxidation with sodium dichromate (Na_2_Cr_2_O_7_ 4N) and sulfuric acid (H_2_SO_4_ 10N). Remaining phosphorus (P‐rem) was measured by equilibrating the soil with a standard P solution.

### Biocrust sampling and identification

In March 2020, the random‐walk method was used to collect biocrust samples by two researchers, who sampled simultaneously for about 2 h per site to ensure even effort. Biocrusts (~100 cm^2^, ≥1 m apart) were harvested from the topsoil using a spatula, placed in paper bags, and transported to the laboratory. A decision tree approach following Weber *et al*. (2022) confirmed the classification of samples as biocrusts, and their composition was analysed using stereomicroscopy and light microscopy. Bryophytes were identified to species level based on Gradstein *et al*. (2001) and associated references, while cyanobacteria were identified to genus level using Komárek & Anagnostidis (1999, 2005) and Komárek (2013). Voucher specimens were deposited in the BHCB herbarium at Universidade Federal de Minas Gerais (Table S1).

### Assessing biocrust' species life strategies

During (1979) was used to classify bryophyte life strategies into fugitives, colonizers, annuals, short‐lived species, perennial shuttle species, and perennial stayers. Fugitives and colonizers grow rapidly, invest heavily in sexual reproduction, have short lifespans, lack asexual reproduction, produce small spores, and exhibit turf life forms, thriving in disturbed environments. In contrast, perennial shuttle species and stayers are found in stable ecosystems, with slower growth, lower reproductive output, long lifespans, large spores, and life forms such as mats, wefts, and dendroids.

Based on During's (1979) framework, we developed a numerical scale to classify bryophyte species. Lower scores correspond to fugitives and colonists, while higher scores indicate perennial shuttle species and stayers. Six traits were evaluated: spore size, asexual reproduction, life form, water uptake adaptations, sexual system, and geographical distribution (Table S2). Each trait was categorized following During (1979) and assigned values of 1, 5, or 10. Species scores were obtained by summing trait values (Table S3). Trait data were sourced from Gradstein *et al*. (2001) and references therein.

Cyanobacteria in biocrusts are categorized into pioneers, biocrust stayers, and stochastic species (Büdel *et al*. 2016). Pioneers are filamentous cyanobacteria that stabilize the soil by binding particles through an extracellular matrix. Biocrust stayers, which can be filamentous or unicellular, play a role in carbon and nitrogen cycling. Stochastic species, originating from other habitats, may establish within biocrusts. Büdel *et al*. (2016) identified several cyanobacterial genera within these groups, and based on this classification, we qualitatively categorized the cyanobacteria at both study sites, without applying a numerical scale.

### Statistical analyses

To explore soil chemical variations, we applied Non‐metric Multidimensional Scaling (NMDS) using the ‘vegan’ package, which facilitated the visualization of patterns among soil properties. Due to the high number of soil variables relative to the sample size, NMDS results were used to select eight key variables for Multivariate ANOVA, ensuring the assumptions of normality and homogeneity were met. MANOVA was then employed to identify the variables driving site differences. Data were log_10_(*x* + 1) transformed to meet the assumptions. A Mann–Whitney *U* test was conducted to compare bryophyte biocrust life strategy scores between sites. Since no differences in cyanobacteria life strategies were observed between sites, statistical analysis was not performed for them. All analyses were conducted in R v. 4.0.2 (R Development Core Team 2020).

## RESULTS

### Soil chemical properties

Soil analysis revealed higher concentrations of K, P, Ca, Mg, Al, and Mn in the preserved site (PS), while the impacted site (IS) had higher levels of Na, Zn, Fe, Cu, and S (Table S4). Boron levels were minimal at both sites (0.01 mg dm^−3^). The pH was slightly acidic, measuring 4.62 in PS and 5.04 in IS. Most soil variables showed lower averages in IS, with the exception of BSI, which remained similar between the sites (Table S4). The preliminary assessment of soil chemical properties at the study sites, conducted using NMDS (Table S5), revealed that PRem, ECEC, P, SB, and Ca were positively correlated with the preserved site and negatively with the impacted site (Fig. S2). Conversely, only S showed a positive correlation with the impacted site and a negative correlation with the preserved site (Fig. S2). Notably, one of the impacted soil samples was an outlier, being the only sample positively correlated with pH, Na, BSI, and Mn (Fig. S2). MANOVA indicated significant differences (*P* < 0.05) in P, S, CEC, and ECEC among the most relevant NMDS variables (Table S6). IS had lower CEC and ECEC than PS (Fig. S3). Additionally, P and S showed contrasting patterns, with higher S in IS and higher P in PS (Fig. S3).

### Species diversity in biocrusts

The biocrust survey resulted in 57 samples: 35 from the PS and 22 from the IS. A total of 132 specimens were recorded, representing three phyla: Bryophyta, Cyanobacteria, and Marchantiophyta (Fig. S4). Mosses dominated the biocrusts, comprising 57.8% of the biocrusts in PS and 69% in IS, with cyanobacteria being the second most abundant group. Liverworts were found exclusively in PS. Both sites shared two moss species (*Bryum orthodontioides* Müll.Hal. and *Fissidens zollingeri* Mont.), and three cyanobacteria genera (*Gloeothec*e Nägeli, *Oscillatoria* Vaucher ex Gomont, and *Scytonema* C.Agardh ex É.Bornet & C.Flahault).

**Fig. 1:**
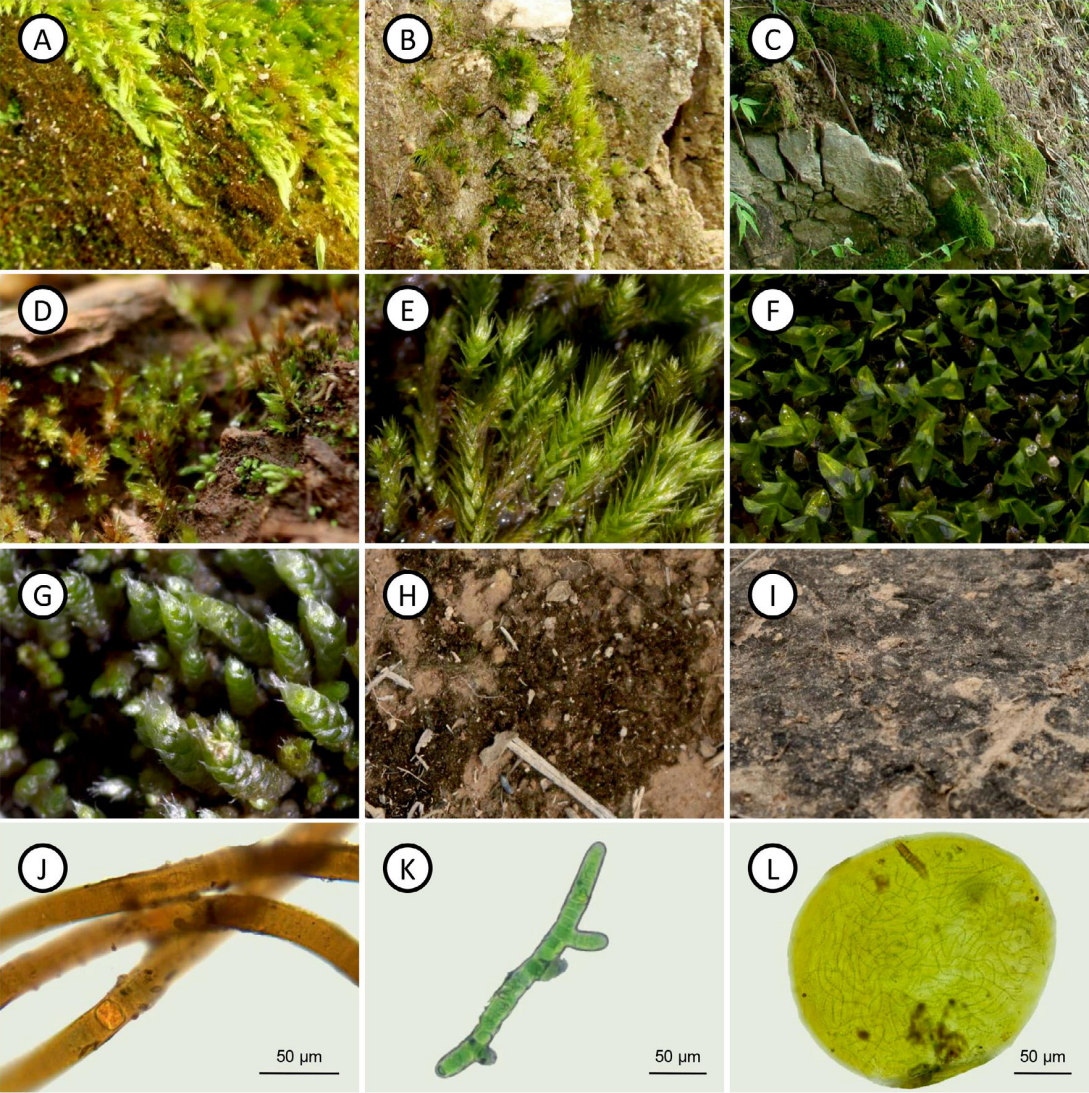
Biocrust species from the study sites. (A) *Vitalia cuspidifera* and *Scytonema* biocrust (preserved site). (B) *Campylopus heterostachys*. (C) *Tortella tortuosa*. (D) *Bryum subapiculatum* biocrust (impacted site). (E) *Philonotis sphaerocarpa*. (F) *Hyophila involuta*. (G) *Bryum argenteum*. (H) *Scytonema*‐dominated biocrust. (I) *Stigonema*‐dominated biocrust. (J) *Scytonema*. (K) *Stigonema*. (L) *Nostoc*.

In the PS, biocrusts were predominantly composed of pleurocarpous mosses, with *Vitalia cuspidifera* (Mitt.) P.E.A.S. Câmara, Carv.‐Silva & W.R. Buck (Fig. 1A) standing out as particularly abundant. However, biocrusts dominated by acrocarpous mosses such as *Campylopus heterostachys* (Hampe) A. Jaeger (Fig. 1B) and *Tortella tortuosa* (Hedw.) Limpr. (Fig. 1C) were also present. In contrast, the IS featured biocrusts with dominance only by acrocarpous mosses, like *Bryum subapiculatum* Hampe (Fig. 1D), *Philonotis sphaerocarpa* (Hedw.) Brid. (Fig. 1E), *Hyophila involuta* (Hook.) A. Jaeger (Fig. 1F), and *Bryum argenteum* Hedw. (Fig. 1G).

In some samples in both studied sites, cyanobacteria were the dominant components. These biocrusts were characterized by their dark coloration and thin structure, measuring only a few millimetres in thickness (Fig. 1H,I). They were primarily composed of filamentous cyanobacteria, such as *Scytonema* C.Agardh ex Bornet & Flahault (Fig. 1J) and *Stigonema* C. Agardh ex Bornet & Flahault (Fig. 1K). Additionally, other genera identified included *Microcoleus* Desmazières ex Gomont, *Nostoc* Vaucher ex Bornet & Flahault (Fig. 1L), *Oscillatoria* Vaucher ex Gomont, *Gloeothece* Nägeli, and *Schizothrix* Kützing ex Gomont.

### Biocrust life strategies

**Fig. 2:**
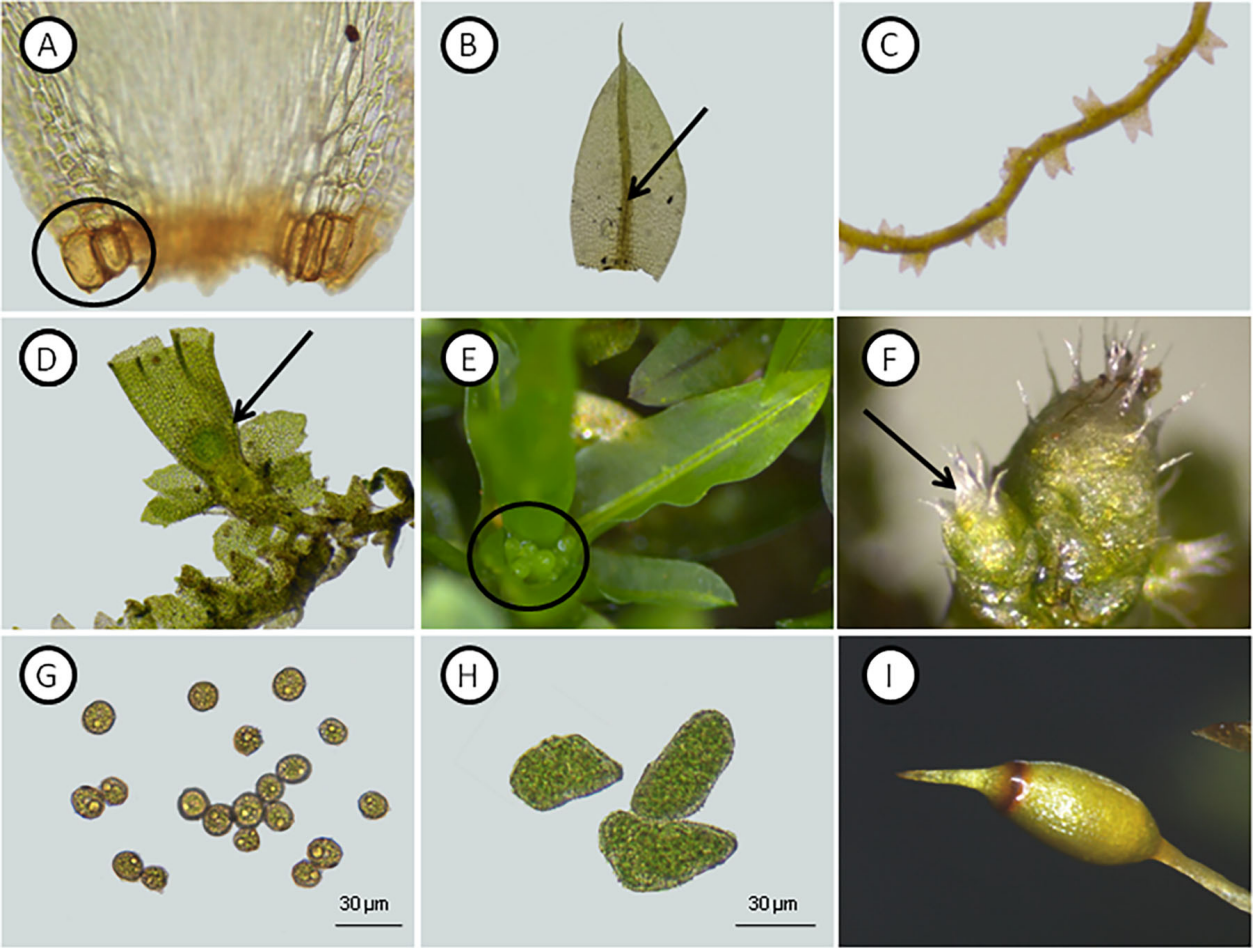
Life traits of bryophyte biocrust species. (A) Alar cells (*Brytonodoxa subpinnata*). (B) Developed costae (*Bryum atenense*). (C) *Cephaloziella granatensis*, a liverwort lacking lobules and underleaves. (D) *Chonecolea doellingeri* sporophyte. (E) Axilary gemmae of *Hyophila involuta*. (F) Bulbils from *Bryum argenteum*. (G) Small spores (*Bryum orthodontioides*). (H) Sporelings (*Cheilolejeunea discoidea*). (I) Sporophyte (*Octoblepharum albidum*).

Bryophyte life traits differed between the sites, reflecting distinct life strategies. In the PS, 50% of bryophytes exhibited mat and weft life forms, while in the IS, 80% displayed a turf life form. Water‐adaptive traits in leaves, such as alar cells (Fig. 2A) and costae (Fig. 2B), were common across both sites, with all IS bryophytes featuring costae. Some species from PS, such as the liverwort *Cephaloziella granatensis* (J.B.Jack) Fulford, lacked efficient water‐capturing structures, notably the absence of lobules (Fig. 2C).

Reproductive strategies also exhibited site‐specific variation. In the PS, 63% of bryophytes were monoicous, frequently producing sporophytes (e.g., *Chonecolea doellingeri* (Nees) Grolle, Fig. 2D). In contrast, 70% of species in the IS were dioicous, relying on asexual propagules such as axillary gemmae (e.g., *H. involuta*, Fig. 2E) and bulbils (e.g., *B. argenteum*, Fig. 2F). Spore size and dispersal capacity also differed: species with small spores, such as *F. zollingeri* and *B. orthodontioides* (Fig. 2G), were found in both sites, whereas species with larger spores and sporelings, like *Cheilolejeunea discoidea* (Lehm. & Lindenb.) Kachr. & R.M. Schust. (Fig. 2H), were restricted to PS. Additionally, species prolific in sporophyte production, such as *Octoblepharum albidum* Hedw. (Fig. 2I), were absent from IS.

**Fig. 3:**
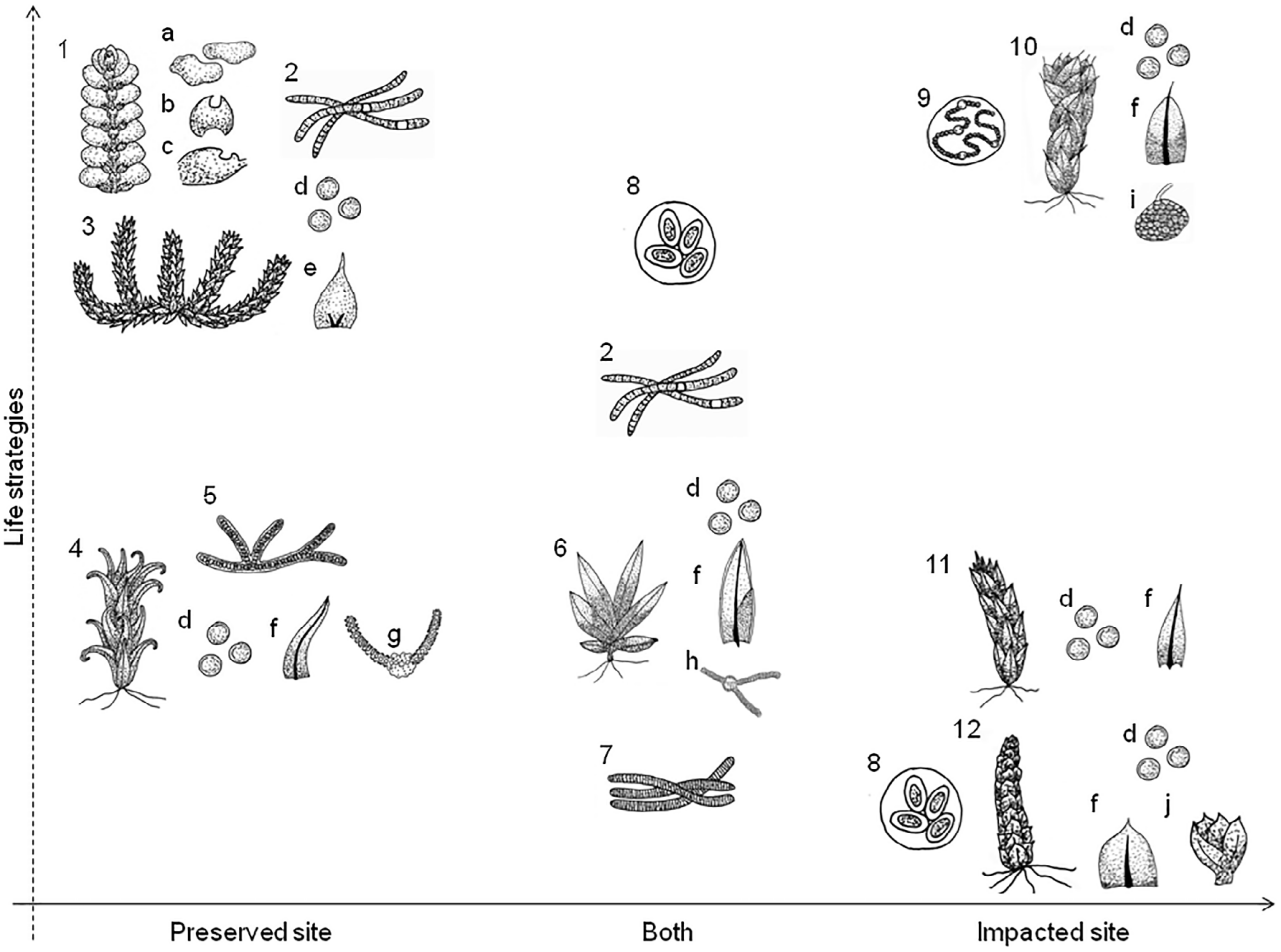
Conceptual model of biocrust species life strategies in preserved and impacted sites along the Gualaxo do Norte River. Species: (1) *Cheilolejeunea discoidea*; (2) *Scytonema*; (3) *Chryso‐hypnum diminutivum*; (4) *Tortella tortuosa*; (5) *Stigonema*; (6) *Fissidens zollingeri*; (7) *Oscillatoria*; (8) *Gloeothece*; (9) *Nostoc*; (10) *Bryum atenense*; (11) *Philonotis sphaerocarpa*; (12) *Bryum argenteum*. Traits: (a) large spores (sporelings); (b) underleaves; (c) lobule; (d) small spores; (e) undeveloped costae; (f) developed costae; (g) leaf papillae; (h) vaginant laminae; (i) tubers; (j) bulbils.

Our life strategy scores highlight *Funaria hygrometrica* Hedw. as the bryophyte species in the studied biocrusts with the lowest value (10; Table S3). Other species, such as *B. argenteum*, *F. zollingeri*, *H. involuta*, *P. sphaerocarpa*, and *T. tortuosa*, also exhibited low values (14). It is important to note that *F. hygrometrica*, *B. argenteum*, *H. involuta*, and *P. sphaerocarpa* were found exclusively in the IS, while *T. tortuosa* was exclusive to the PS, and *F. zollingeri* was present in both study areas. In contrast, the liverworts *C. discoidea* and *Fossombronia porphyrorhiza* (Nees) Prosk. from the PS, along with *Bryum atenense* Williams from the IS, displayed the highest values within our life strategy scores (>30; Table S3). Notably, the Mann–Whitney *U* test revealed a significant difference in life strategies between the two sites (*z*‐score = 1.669; *P* = 0.047). As hypothesized, life strategies differ between IS and PS, with a predominance of pioneer species in the IS and perennial species in the PS (Fig. 3).

Interestingly, the life strategies of cyanobacteria did not differ between the two studied sites. Both sites exhibited equal proportions of pioneer (20%), biocrust‐stayer (40%), and stochastic (40%) species. We categorized *Schizothrix* and *Microcoleus* as pioneer genera, each exclusive to one of the study areas (IS and PS, respectively). The biocrust‐stayer genera included *Nostoc*, *Stigonema*, and *Scytonema*, always presenting heterocysts. *Nostoc* was exclusive to IS, *Stigonema* to PS, and *Scytonema* was abundantly present in both studied sites. Lastly, stochastic genera such as *Gloeothece* and *Oscillatoria* were recorded in both study areas, always epiphytic on bryophytes. Curiously, we found that the same cyanobacterial taxa were present with bryophytes spanning a wide range of life strategy scores (Fig. 3).

## DISCUSSION

### Soil conditions and biocrust species diversity

Our soil analyses revealed distinct conditions between the study sites, consistent with previous research on Fundão Dam tailings (Santos *et al*. 2019; Scotti *et al*. 2020; Couto *et al*. 2021). However, key characteristics, such as alkaline pH and high Na concentrations, were absent in the IS. The pH of the tailings (5.04) was similar to that of the PS (4.72), which aligns with reports of alkaline tailings (pH ~8; Santos *et al*. 2019) becoming more acidic over time (~6.5; Couto *et al*. 2021), likely due to microbial activity and vegetation establishment (Gomes *et al*. 2021), with biocrusts potentially contributing to this shift (Fan *et al*. 2023). The elevated Na levels in IS were caused by a single high‐Na sample, suggesting localized contamination, as noted in previous studies (Santos *et al*. 2019; Scotti *et al*. 2020), although it was not sufficient to create a significant difference between the sites.

High Na levels can be toxic to many organisms, especially plants (Kronzucker *et al*. 2013). However, some biocrust components, particularly bryophytes, exhibit adaptive mechanisms to tolerate salinity (Wang *et al*. 2008). For example, *F. hygrometrica*, found exclusively in IS, produces protein kinases that mitigate cellular damage and detoxify harmful compounds (D'Souza & Johri 2002). Likewise, *Bryum* species, the most abundant mosses in IS, are known for their salinity tolerance (Garbary *et al*. 2008; Lobachevska *et al*. 2019), aiding their persistence despite localized Na contamination in the IS.

Our results also indicate significant differences in P and S levels between sites, with IS exhibiting higher S and lower P concentrations. This imbalance may stem from disrupted biological functions and microbial nutrient cycling, which are often affected by mining tailings (Prado *et al*. 2023). However, Scotti *et al*. (2020), studying a nearby area close to our study sites, found no significant P and S differences between natural soils and mining tailings. This discrepancy suggests that while mining tailings can disrupt biological functions and nutrient cycling, natural variability between preserved sites may also influence P and S levels. Both CEC and ECEC were significantly lower in IS, indicating reduced fertility and nutrient retention (Jones & Jacobsen 2005), a pattern commonly observed in Fundão Dam tailings (Silva *et al*. 2022).

These soil factors likely influence biocrust species diversity, as biocrust cover, richness, and composition strongly correlate with soil properties (Bowker *et al*. 2016). Low P concentrations are often negatively associated with biocrusts (Bowker *et al*. 2005, 2006), although biocrust organisms can incorporate P into their biomass, preventing its adsorption onto mineral surfaces (Crain *et al*. 2018), possibly explaining the low P levels beneath biocrusts. Excessive S can impair plant growth by causing oxidative stress, cellular damage, and chlorophyll degradation (Cui & Wang 2002), although Bryaceae mosses exhibit adaptability to high S levels (Han *et al*. 2022). This family was the most represented in IS, suggesting tolerance to these conditions.

Biocrusts are frequently found in mining tailings, with mosses as dominant components (Liao, Chen, *et al*. 2024), particularly Bryaceae and Pottiaceae, which tolerate extreme pH, nutrient deficiency, and drought (Seppelt *et al*. 2016). Among mosses, the genera *Bryum* and *Barbula* are most commonly found in tailings (Liao, Chen, *et al*. 2024). In our study, we identified four *Bryum* species in IS, along with some Pottiaceae representatives. In contrast, although the cyanobacterial genus *Nostoc* is the most frequently reported within biocrusts (Dal‐Ferro *et al*. 2024), including in mining tailings globally (Liao, Chen, *et al*. 2024), we detected it in only one sample.

### Life strategies of biocrust organisms

As hypothesized, life strategies differed between sites, notably in bryophytes. Many IS mosses exhibited fugitive and colonizer traits (During 1979), while perennial traits were prevalent in PS. Turf life forms, known for superior desiccation tolerance (Glime 2017), were present in all IS mosses, whereas PS biocrusts featured mat and weft forms, typically associated with perennial strategies (During 1979, 1992). Turf mosses thrive in high sunlight and low humidity (During 1979; Glime 2017), conditions likely intensified in IS due to vegetation loss (Cruz *et al*. 2020). To cope with these stresses, IS mosses exhibited traits enhancing water absorption and transport, such as costae, alar cells, hyaline hair‐points, hyalocysts, and papillae (Watson 1914; Glime 2017). Notably, all IS bryophytes had costae, whereas some PS species lacked structures aiding water capture.

Monoicous bryophytes typically produce more sporophytes (Longton & Schuster 1983; Maciel‐Silva & Pôrto 2014), whereas dioicous species compensate by relying on asexual propagules (Longton & Schuster 1983; Glime 2017). In IS, dioicous species such as *B. argenteum* and *H. involuta* produced abundant asexual propagules, specifically bulbils and gemmae, respectively. These structures play a key role in early colonization (During 1979), further supporting the dominance of colonist species in IS. Although we did not quantify sporophyte production, we observed that dioicous specimens collected in IS generally lacked sporophytes.

Spore size and dispersal capacity also influenced bryophyte distributions. Small‐spored species, like *F. zollingeri* and *B. orthodontioides*, were present in both sites, likely dispersing via warm air currents (Frahm 2007). Conversely, large‐spored species, such as PS liverworts, faced dispersal limitations (During 1979) and lacked adaptations to IS conditions, restricting their establishment (Lynch & Gabriel 1987; Chapin III *et al*. 1993). Similarly, species prolific in sporophyte production in PS, such as *O. albidum*, did not colonize IS, as habitat changes impact colonization success (Keddy 1992; Lavorel & Garnier 2002).

Fugitive and colonist strategies often dominate mining tailings (Širka *et al*. 2019). The most common fugitive moss *F. hygrometrica*, thrives in burnt and mining sites, producing numerous sporophytes (Shaw 1987), aligning with its presence in IS. Another common pioneer, *B. argenteum*, initiates bryophyte cover in disturbed areas (Lobachevska *et al*. 2019). Some PS species with lower life strategy scores, like *T. tortuosa*, have also been recorded in mining sites (Širka *et al*. 2019). These findings reinforce that fugitive and colonist traits – high dispersal, rapid growth, and stress tolerance – facilitate bryophyte establishment in degraded environments, whereas perennial species, dependent on stability, struggle to colonize disturbed sites (During 1979).

Although cyanobacterial life strategies did not differ between sites, diversity did. However, functionally similar taxa played comparable ecological roles. Pioneer genera (*Microcoleus* and *Schizothrix*) contributed to early soil stabilization (Büdel *et al*. 2016), while biocrust stayers (*Scytonema*, *Stigonema* and *Nostoc*) supported nitrogen fixation (Barger *et al*. 2016; Büdel *et al*. 2016). Stochastic genera (*Gloeothece* and *Oscillatoria*), epiphytic on bryophytes, likely engaged in commensal or mutualistic interactions. Bryophytes attract heterocystous cyanobacteria via chemical signals, exchanging carbon and sulfur for fixed nitrogen (Stuart *et al*. 2020). Since *Gloeothece* and *Oscillatoria* lack heterocysts, their presence suggests a commensal relationship.

### Insights from biocrust life strategies for enhancing restoration efforts

Biocrusts are well‐established in landscape restoration (Antoninka *et al*. 2020; Malešević *et al*. 2024), yet their application in mining tailings remains limited (Liao, Chen, *et al*. 2024). Some cyanobacterial studies focus on inoculating *Nostoc* sp. (Rezasoltani *et al*. 2024) and *Oscillatoria* sp. (Zanganeh *et al*. 2022) in others types of tailings, suggesting restoration potential. *Oscillatoria* sp. aids soil improvement, heavy metal immobilization, and plant growth (Zanganeh *et al*. 2022), while *Nostoc* sp. enhances soil properties, produces extracellular polymeric substances, and strengthens surface stability (Rezasoltani *et al*. 2024). These effects could support Fundão Dam tailings rehabilitation.

Few and recent studies have explored moss‐dominated biocrust inoculation in mining tailings (Liao, Tao, *et al*. 2024; Oliveira *et al*. 2024). The species tested, *Pottia truncatula* [L.] Lindb. (Liao, Tao, *et al*. 2024) and *H. involuta* (Oliveira *et al*. 2024), belong to Pottiaceae, a family adapted to harsh environments (Zander 1993). Bryophytes suitable for restoration typically develop asexual propagules (axillary buds, bulbils, gemmae), produce abundant spores, and exhibit a monoicous system (Rosentreter 2020). In our study, *F. hygrometrica*, *B. argenteum*, and *H. involuta* emerge as promising candidates due to their low life strategy scores, indicating strong establishment potential in degraded conditions.

Biocrust inoculation in Fundão Dam tailings could be effective, as many species already present exhibit adaptations to adverse conditions and contribute to surface soil restoration. The lower P concentration in IS may be mitigated by biocrusts, which enhance P bioavailability (Baumann *et al*. 2018). Biocrusts also increase soil organic content, improving cation exchange capacity (Guo *et al*. 2008; Zhao *et al*. 2014), and facilitate carbon and nitrogen fixation (Barger *et al*. 2016; Sancho *et al*. 2016), promoting soil organic matter accumulation (Baumann *et al*. 2021). Additionally, they enhance soil aggregation, stabilization (Belnap & Büdel 2016), and moisture retention (Chamizo *et al*. 2016), underscoring their key role in mining tailings restoration.

## CONCLUSIONS

Our results indicate that while some soil parameters in mining tailings are approaching pre‐disaster conditions, others, such as S, P, CEC, and ECEC, remain highly unbalanced. Biocrust organisms play a key role in colonizing disturbed sites, improving soil conditions through nitrogen fixation, water retention, and soil structure enhancement, making them promising organisms for restoration. Mosses from Bryaceae, Pottiaceae, and Funariaceae tolerate abiotic stressors and exhibit life traits that support biocrust persistence in harsh environments. Integrating moss‐dominated biocrusts with cyanobacteria into restoration efforts could enhance soil health, biodiversity, and long‐term sustainability in landscapes affected by the Fundão Dam collapse.

## AUTHOR CONTRIBUTIONS

MFO and ASM‐S conceived and designed the study. All authors contributed to the development of the methodology, data collection, and data analysis. MFO wrote the first draft of the manuscript. All authors critically revised and approved the final version.

## CONFLICT OF INTEREST STATEMENT

The authors declare that they have no known competing financial interests or personal relationships that could have appeared to influence the work reported in this paper.

## Supporting information


**Fig. S1.** Study sites in Paracatu de Baixo, Brazil. (A) Boundary between forest and pasture in the preserved site. (B) Atlantic Forest remnant in the preserved site. (C) Impacted site with abundant grasses and exposed soil dominated by biocrusts. (D) Gualaxo do Norte River near the impacted site.

**Fig. S2.** NMDS analysis of soil variables at the preserved and impacted sites. Arrows indicate significant correlations (*P* ≤ 0.05).

**Fig. S3.** Box plots of significant soil differences between preserved and impacted sites. Lines in boxes: medians; box ends: quartiles; whiskers: range; black dots: means; empty dots: outliers.

**Fig. S4.** Abundance of bryophytes and cyanobacteria in biocrusts from preserved and impacted sites. Liverworts were exclusive to the preserved site. Abundance represents the number of samples where each taxon was recorded.

**Table S1.** Voucher specimens deposited in the BHCB herbarium comprising biological soil crusts collected from both mining tailings and preserved sites within the region affected by the Fundão Dam disaster. IS, impacted site; PS, preserved site.

**Table S2.** Traits related to bryophyte life strategies, with respective assigned values for categories and description.

**Table S3.** Bryophytes species collected in the mining tailing and preserved site, and their unique values for life strategies based on the sum of trait scores. PS, preserved site; IS, impacted site; BS, both sites; SS, spore size; AR, asexual reproduction; LF, life form; WUA, water uptake adaptations; Sex, sexual system; GD, geographical distribution. *Based on genus information.

**Table S4.** Descriptive results of chemical properties for each sampled area. SD, standard deviation.

**Table S5.** Site scores on the first and second components of the NMDS based on soil variables. In bold, the most significant variables with *P*‐values < 0.05 in the NMDS, and with a * those that were used in the MANOVA.

**Table S6.** Summary of MANOVA for the soil variables across the study sites. Significant *P*‐values are in bold; df, degrees of freedom.

## References

[plb70037-bib-0001] Antoninka A. , Faist A. , Rodriguez‐Caballero E. , Young K.E. , Chaudhary V.B. , Condon L.A. , Pyke D.A. (2020) Biological soil crusts in ecological restoration: emerging research and perspectives. Restoration Ecology, 28, S3–S8.

[plb70037-bib-0002] Barger N.N. , Weber B. , Garcia‐Pichel F. , Zaady E. , Belnap J. (2016) Patterns and controls on nitrogen cycling of biological soil crusts. In: Weber B. , Büdel B. , Belnap J. (Eds), Biological soil crusts: an organizing principle in drylands. Springer, Cham, Switzerland, pp 257–285.

[plb70037-bib-0003] Baumann K. , Eckhardt K. , Acksel A. , Gros P. , Glaser K. , Gillespie A.W. , Karsten U. , Leinweber P. (2021) Contribution of biological soil crusts to soil organic matter composition and stability in temperate forests. Soil Biology and Biochemistry, 160, 108315.

[plb70037-bib-0004] Baumann K. , Jung P. , Samolov E. , Lehnert L.W. , Büdel B. , Karsten U. , Bendix J. , Achilles S. , Schermer M. , Matus F. , Oses R. , Osses P. , Morshedizad M. , Oehlschläger C. , Hu Y. , Klysubun W. , Leinweber P. (2018) Biological soil crusts along a climatic gradient in Chile: richness and imprints of phototrophic microorganisms in phosphorus biogeochemical cycling. Soil Biology and Biochemistry, 127, 286–300.

[plb70037-bib-0005] Belnap B. , Büdel B. (2016) Biological soil crusts as soil stabilizers. In: Weber B. , Büdel B. , Belnap J. (Eds), Biological soil crusts: an organizing principle in drylands. Springer, Cham, Switzerland, pp 305–320.

[plb70037-bib-0006] Bowker M.A. , Belnap J. , Büdel B. , Sannier C. , Pietrasiak N. , Eldridge D.J. , Rivera‐Aguilar V. (2016) Controls on distribution patterns of biological soil crusts at micro‐ to global scales. In: Weber B. , Büdel B. , Belnap J. (Eds), Biological soil crusts: an organizing principle in drylands. Springer, Cham, Switzerland, pp 55–80.

[plb70037-bib-0007] Bowker M.A. , Belnap J. , Davidson D.W. , Goldstein H. (2006) Correlates of biological soil crust abundance across a continuum of spatial scales: support for a hierarchical conceptual model. Journal of Applied Ecology, 43, 152–163.

[plb70037-bib-0008] Bowker M.A. , Belnap J. , Davidson D.W. , Phillips S.L. (2005) Evidence for micronutrient limitation of biological soil crusts: importance to arid‐lands restoration. Ecological Applications, 15, 1941–1951.

[plb70037-bib-0009] Büdel B. , Dulic T. , Darienko T. , Rybalka N. , Friedl T. (2016) Cyanobacteria and algae of biological soil crusts. In: Weber B. , Büdel B. , Belnap J. (Eds), Biological soil crusts: an organizing principle in drylands. Springer, Cham, Switzerland, pp 55–80.

[plb70037-bib-0010] Carmo F.F. , Kamino L.H.Y. , Junior R.T. , Campos I.C. , Carmo F.F. , Silvino G. , Castro K.J.S.X. , Mauro M.L. , Rodrigues N.U.A. , Miranda M.P.S. , Pinto C.E.F. (2017) Fundão tailings dam failures: the environment tragedy of the largest technological disaster of Brazilian mining in global context. Perspectives in Ecology and Conservation, 15, 145–151.

[plb70037-bib-0011] Chamizo S. , Belnap J. , Eldridge D.J. , Cantón Y. , Issa O.M. (2016) The role of biocrusts in arid land hydrology. In: Weber B. , Büdel B. , Belnap J. (Eds), Biological soil crusts: an organizing principle in drylands. Springer, Cham, Switzerland, pp 321–346.

[plb70037-bib-0012] Chapin F.S., III , Autumn K. , Pugnaire F. (1993) Evolution of suites of traits in response to environmental stress. The American Naturalist, 142, S78–S92.

[plb70037-bib-0013] Claessen M.E.C. , Barreto W.O. , Paula J.L. , Duarte M.N. (1997) Manual de métodos de análise de solo. EMBRAPA‐CNPS, Rio de Janeiro, Brazil, pp 212.

[plb70037-bib-0014] Couto F.R. , Ferreira A.M. , Pontes P.P. , Marques A.R. (2021) Physical, chemical and microbiological characterization of the soils contaminated by iron ore tailing mud after Fundão dam disaster in Brazil. Applied Soil Ecology, 158, 103811.

[plb70037-bib-0015] Crain G.M. , McLaren J.R. , Brunner B. , Darrouzet‐Nardi A. (2018) Biologically available phosphorus in biocrust‐dominated soils of the Chihuahuan Desert. Soil Systems, 2, 56.

[plb70037-bib-0016] Cruz F.V.S. , Gomes M.P. , Bicalho E.M. , Della Torre F. , Garcia Q.S. (2020) Does Samarco's spilled mud impair the growth of native trees of the Atlantic rainforest? Ecotoxicology and Environmental Safety, 189, 110021. 31830604 10.1016/j.ecoenv.2019.110021

[plb70037-bib-0017] Cui Y.S. , Wang Q.R. (2002) Sulfur behavior in soil and atmosphere environment and its effect on plants. Chinese Journal of Eco‐Agriculture, 10, 80–82.

[plb70037-bib-0018] Dal‐Ferro L.S. , Schenider A. , Missiaggia D.G. , Silva L.J. , Maciel‐Silva A.S. , Figueredo C.C. (2024) Organizing a global list of cyanobacteria and algae from soil biocrusts evidenced great geographic and taxonomic gaps. FEMS Microbiology Ecology, 100, fiae086. 38816216 10.1093/femsec/fiae086 PMC11221558

[plb70037-bib-0019] D'Souza J.S. , Johri M.M. (2002) ABA and NaCl activate myelin basic protein kinase in the chloronema cells of the moss *Funaria hygrometrica* . Plant Physiology and Biochemistry, 40, 17–24. 10.1016/S0981-9428(01)01344-4

[plb70037-bib-0020] During H.J. (1979) Life strategies of bryophytes: a preliminary review. Lindbergia, 5, 2–18.

[plb70037-bib-0021] During H.J. (1992) Ecological classifications of bryophytes and lichens. In: Bates J.W. , Farmer A.M. (Eds), Bryophytes and lichens in a changing environment. Clarendon Press, Oxford, UK, pp 1–31.

[plb70037-bib-0022] Fan J. , Bu C. , Qi Y. , Zhou W. , Wang C. , Wei Y. , Siddique K.H.M. (2023) Biocrusts significantly affect the bioavailability and ecological risk of heavy metals in gold mine tailings. Plant and Soil, 493, 99–113.

[plb70037-bib-0023] Frahm J.P. (2007) Diversity, dispersal and biogeography of bryophytes (mosses). In: Foissner W. , Hawksworth D.L. (Eds), Protist diversity and geographical distribution. Springer, Dordrecht, Netherlands, pp 43–50.

[plb70037-bib-0024] Garbary D.J. , Miller A.G. , Scrosati R. , Kim K.Y. , Schofield W.B. (2008) Distribution and salinity tolerance of intertidal mosses from Nova Scotian salt marshes. The Bryologist, 111, 282–291.

[plb70037-bib-0025] Glime J.M. (2017) Bryophyte ecology. Michigan Technological University and the International Association of Bryologists, Houghton, USA.

[plb70037-bib-0026] Gomes A.R. , Antão A. , Santos A.G.P. , Lacerda T.J. , Medeiros M.B. , Saenz L.A.I. , Alvarenga S. , Santos C.H. , Rigobelo E.C. , Muzzi M.R.S. (2021) Rehabilitation of a riparian site contaminated by tailings from the Fundão dam, Brazil, using different remediation strategies. Environmental Toxicology and Chemistry, 40, 2359–2373. 33928667 10.1002/etc.5075

[plb70037-bib-0027] Gomes A.R. , Antão A. , Santos C.H. , Rigobelo E.C. , Scotti M.R. (2024) Assessing the reclamation of a contaminated site affected by the Fundão dam tailings trough phytoremediation and bioremediation. International Journal of Phytoremediation, 26, 1305–1320. 38391288 10.1080/15226514.2024.2315471

[plb70037-bib-0028] Gradstein S.R. , Churchill S.P. , Allen N.S. (2001) Guide to the bryophytes of tropical america. Memoirs of the New York Botanical Garden, 2001, 86: I–VIII, 1–577, 2219 fig. auteur: Systematisch‐geobotaniches Institut, Univ. Göttingen, Untere Karspüle 2, D‐73073 Göttingen; éditeur: NYBG Press, Bronx River Parkway at Fordham Road, Bronx, New Yo…. Cryptogamie Bryologie.

[plb70037-bib-0029] Green T.G.A. , Proctor M.C.F. (2016) Physiology of photosynthetic organisms within biological soil crusts: their adaptation, flexibility, and plasticity. In: Belnap J. , Weber B. , Büdel B. (Eds), Biological soil crusts: an organizing principle in drylands. Springer, Cham, Switzerland, pp 347–381.

[plb70037-bib-0030] Guo Y. , Zhao H. , Zuo X. , Drake S. , Zhao X. (2008) Biological soil crust development and its topsoil properties in the process of dune stabilization, Inner Mongolia, China. Environmental Geology, 54, 653–662.

[plb70037-bib-0031] Han J.H. , Zhang Z.H. , Wang Z.H. (2022) Responses of acrocarpous moss communities to heavy metal (Fe, Mn, Cd) and sulfur pollution in the Changgou carbonate manganese ore, SW China. Journal of Mountain Science, 19, 1292–1306.

[plb70037-bib-0032] Jones C. , Jacobsen J. (2005) Plant nutrition and soil fertility. Nutrient Management Module, 2, 1–11.

[plb70037-bib-0033] Keddy P.A. (1992) Assembly and response rules: two goals for predictive community ecology. Journal of Vegetation Science, 3, 157–164.

[plb70037-bib-0034] Komárek J. (2013) Cyanoprokaryota 3. Teil: Heterocytous genera. In: Büdel B. , Gärtner G. , Krienitz L. , Schagerl M. (Eds), Süβwasserflora von Mitteleuropa 19/3. Springer, Berlin, Germany, pp 1130.

[plb70037-bib-0035] Komárek J. , Anagnostidis K. (1999) Cyanoprokaryota, 1: Chroococcales. In: Ettl H. , Gärtner G. , Heynig H. , Mollenheuer D. (Eds), Süsswasserflora von Mitteleurope. Springer, Berlin, Germany, pp 1–548.

[plb70037-bib-0036] Komárek J. , Anagnostidis K. (2005) Cyanoprokaryota 1. Teil: Oscillatoriales. In: Büdel B. , Krienitz L. , Gärtner G. , Schagerl M. (Eds), Süβwasserflora von Mitteleuropa 19/2. Elsevier/Spektrum, Berlin, Germany, pp 759.

[plb70037-bib-0037] Kronzucker H.J. , Coskun D. , Schulze L.M. , Wong J.R. , Britto D.T. (2013) Sodium as nutrient and toxicant. Plant and Soil, 369, 1–23.

[plb70037-bib-0038] Lavorel S. , Garnier E. (2002) Predicting changes in community composition and ecosystem functioning from plant traits: Revisiting the holy grail. Functional Ecology, 16, 545–556.

[plb70037-bib-0039] Liao K. , Chen C. , Ye W. , Zhu J. , Li Y. , She S. , Wang P. , Tao Y. , Lv A. , Wang X. , Chen L. (2024) The adaptability, distribution, ecological function and restoration application of biological soil crusts on metal tailings: A critical review. Science of the Total Environment, 927, 172169. 38582126 10.1016/j.scitotenv.2024.172169

[plb70037-bib-0040] Liao K. , Tao Y. , Tu J. , Zeng Y. , Li Y. , Wang P. , Li X. , He F. , Chen L. (2024) Induced and natural moss soil crusts accelerate the C, N, and P cycles of Pb‐Zn tailings. Science of the Total Environment, 909, 168657. 37979864 10.1016/j.scitotenv.2023.168657

[plb70037-bib-0041] Lobachevska O.V. , Kyyak N.Y. , Rabyk I.V. (2019) Ecological and physiological peculiarities of bryophytes on a post‐technogenic salinized territory. Biosystems Diversity, 27, 342–348.

[plb70037-bib-0042] Longton R.E. , Schuster R.M. (1983) Reproductive biology. In: Schuster R.M. (Ed), New manual of bryology. The Hattori Botanical Laboratory, Nichinan, Japan, pp 386–462.

[plb70037-bib-0043] Lynch M. , Gabriel W. (1987) Environmental tolerance. The American Naturalist, 129, 283–303. 10.1086/432558 16224689

[plb70037-bib-0044] Maciel‐Silva A.S. , Pôrto K.C. (2014) Reproduction in bryophytes. In: Ramawat K.G. , Merillon J.M. , Shivanna K.R. (Eds), Reproductive biology of plants. CRC Press, Boca Raton, USA, pp 57–81.

[plb70037-bib-0045] Malešević T.P. , Meriluoto J. , Mihalj I. , Važić T. , Marković R. , Jurca T. , Codd G.A. , Svirčev Z. (2024) Restoration of damaged drylands through acceleration of biocrust development. Catena, 244, 108265.

[plb70037-bib-0046] Oliveira M.F. , Santos P.O. , Oliveira G.F. , Trajano G.O. , Maciel‐Silva A.S. (2024) A promising application of biodegradable glue to improve the efficiency of biocrust inoculation on mining tailings in Brazil. Restoration Ecology, 33, e14365.

[plb70037-bib-0047] Prado I.G.O. , Veloso T.G.R. , Luz J.M.R. , Silva M.C.S. , Prado D.G.O. , Parsons W.F.J. , Khasa D.P. , Kasuya M.C.M. (2023) Total and arbuscular mycorrhizal fungal communities in the first 3 years after the collapse of the Fundão dam: Are we on the ecosystem recovery pathway? Restoration Ecology, 31, e13954.

[plb70037-bib-0048] R Development Core Team (2020) R: A language and environment for statistical computing. R Foundation for Statistical Computing, Vienna, Austria.

[plb70037-bib-0049] Rezasoltani S. , Champagne P. , Mann V. (2024) Improvement in mine tailings biophysicochemical properties by means of cyanobacterial inoculation. Waste and Biomass Valorization, 15, 1689–1699.

[plb70037-bib-0050] Rosentreter R. (2020) Biocrust lichen and moss species most suitable for restoration projects. Restoration Ecology, 28, S67–S74.

[plb70037-bib-0051] Sancho L.G. , Belnap J. , Colesie C. , Raggio J. , Weber B. (2016) Carbon budgets of biological soil crusts at micro‐, meso‐, and global scales. In: Weber B. , Büdel B. , Belnap J. (Eds), Biological soil crusts: An organizing principle in drylands. Springer, Cham, Switzerland, pp 287–304.

[plb70037-bib-0052] Santos G.S. , Silva E.E.C. , Barroso G.F. , Pasa V.M.D. , Eskinazi‐Sant'Anna E.M. (2022) Do metals differentiate zooplankton communities in shallow and deep lakes affected by mining tailings? The case of the Fundão dam failure (Brazil). Science of the Total Environment, 806, 150493. 34844302 10.1016/j.scitotenv.2021.150493

[plb70037-bib-0053] Santos H.G. , Jacomine P.K.T. , Anjos L.H.C. , Oliveira V.A. , Lumbreras J.F. , Coelho M.R. , Almeida J.A. , Filho J.C.A. , Oliveira J.B. , Cunha T.J.F. (2018) Sistema brasileiro de classificação de solos. EMBRAPA‐CNPS, Rio de Janeiro, Brazil, pp 357.

[plb70037-bib-0054] Santos O.S.H. , Avellar F.C. , Alves M. , Trindade R.C. , Menezes M.B. , Ferreira M.C. , França G.S. , Cordeiro J. , Sobreira F.G. , Yoshida I.M. , Moura P.M. , Baptista M.B. , Scotti M.R. (2019) Understanding the environmental impact of a mine dam rupture in Brazil: Prospects for remediation. Journal of Environmental Quality, 48, 439–449. 30951136 10.2134/jeq2018.04.0168

[plb70037-bib-0055] Scotti M.R. , Gomes A.R. , Lacerda T.J. , Ávila S.S. , Silva S.L. , Antão A. , Santos A.G.P. , Medeiros M.B. , Alvarenga S. , Santos C.H. , Rigobelo E.C. (2020) Remediation of a riparian site in the Brazilian Atlantic forest reached by contaminated tailings from the collapsed Fundão dam with native woody species. Integrated Environmental Assessment and Management, 16, 669–675. 32196962 10.1002/ieam.4272

[plb70037-bib-0056] Seppelt R.D. , Downing A.J. , Deane‐Coe K.K. , Zhang Y. , Zhang J. (2016) Bryophytes within biological soil crusts. In: Weber B. , Büdel B. , Belnap J. (Eds), Biological soil crusts: An organizing principle in drylands. Springer, Cham, Switzerland, pp 101–120.

[plb70037-bib-0057] Shaw J. (1987) Effect of environmental pretreatment on tolerance to copper and zinc in the moss *Funaria hygrometrica* . American Journal of Botany, 74, 1466–1475.

[plb70037-bib-0058] Silva A.P.V. , Silva A.O. , Lima F.R.D. , Benedet L. , Franco A.J. , Souza J.K. , Ribeiro Júnior A.C. , Batista É.R. , Inda A.V. , Curi N. , Guilherme L.R.G. , Carneiro M.A.C. (2022) Potentially toxic elements in iron mine tailings: Effects of reducing soil pH on available concentrations of toxic elements. Environmental Research, 215(Pt 2), 114321. 10.1016/j.envres.2022.114321 36222244

[plb70037-bib-0059] Singh H. (2018) Desiccation and radiation stress tolerance in cyanobacteria. Journal of Basic Microbiology, 58, 813–826. 30080267 10.1002/jobm.201800216

[plb70037-bib-0060] Širka P. , Galvánek D. , Turisová I. , Sabovljević M. (2019) What are the main drivers affecting the pattern of bryophyte life history traits at two contrasting spoil heaps? Flora, 253, 17–26.

[plb70037-bib-0061] Stuart R.K. , Pederson E.R.A. , Weyman P.D. , Weber P.K. , Rassmussen U. , Dupont C.L. (2020) Bidirectional C and N transfer and a potential role for sulfur in an epiphytic diazotrophic mutualism. The ISME Journal, 14, 3068–3078. 32814866 10.1038/s41396-020-00738-4 PMC7784912

[plb70037-bib-0062] Vitt D.H. , Crandall‐Stotler B. , Wood A.J. , Rajakaruna N. , Boyd R.S. , Harris T.B.V. (2014) Plant ecology and evolution in harsh environments. Nova Science, New York, USA, pp 267–295.

[plb70037-bib-0063] Wang X. , Liu Z. , He Y. (2008) Responses and tolerance to salt stress in bryophytes. Plant Signaling & Behavior, 3, 516–518. 19513243 10.4161/psb.3.8.6337 PMC2634484

[plb70037-bib-0064] Watson W. (1914) Xerophytic adaptations of bryophytes in relation to habitat. New Phytologist, 13, 149–169.

[plb70037-bib-0065] Weber B. , Belnap J. , Büdel B. , Antoninka A.J. , Barger N.N. , Chaudhary V.B. , Darrouzet‐Nardi A. , Eldridge D.J. , Faist A.M. , Ferrenberg S. , Havrilla C.A. , Huber‐Sannwald E. , Issa O.M. , Maestre F.T. , Reed S.C. , Rodriguez‐Caballero E. , Tucker C. , Young K.E. , Zhang Y. , Zhao Y. , Zhou X. , Bowker M.A. (2022) What is a biocrust? A refined, contemporary definition for a broadening research community. Biological Reviews, 97, 1768–1785. 35584903 10.1111/brv.12862 PMC9545944

[plb70037-bib-0066] Zander R.H. (1993) Genera of the Pottiaceae: Mosses of harsh environments. Bulletin of the Buffalo Society of Natural Sciences, New York, USA.

[plb70037-bib-0067] Zanganeh F. , Heidari A. , Sepehr A. , Rohani A. (2022) Bioaugmentation and bioaugmentation–assisted phytoremediation of heavy metal contaminated soil by a synergistic effect of cyanobacteria inoculation, biochar, and purslane (*Portulaca oleracea* L.). Environmental Science and Pollution Research, 29, 6040–6059. 34432211 10.1007/s11356-021-16061-0

[plb70037-bib-0068] Zhao Y. , Zhu Q. , Li P. , Zhao L. , Wang L. , Zheng X. , Ma H. (2014) Effects of artificially cultivated biological soil crusts on soil nutrients and biological activities in the loess plateau. Journal of Arid Land, 6, 742–752.

