# Supplementary material for: Diversity and life strategies of cyanobacteria and bryophytes within biocrusts in the context of mining tailings disasters in Brazil
Source: Plant Biol (Stuttg). 2025 May 9;27(6):1128–36. doi: 10.1111/plb.70037 (PMC12477303; doi:10.1111/plb.70037)
Supplement: Supplementary file 5 — Table S1. Voucher specimens deposited in the BHCB herbarium comprising biological soil crusts collected from both mining tailings and preserved sites within the region affected by the Fundão Dam disaster. IS, impacted site; PS, preserved site. [file PLB-27-1128-s004.docx]

**Table S1 -** Voucher specimens deposited in the BHCB herbarium comprising biological soil crusts collected from both mining tailings and preserved sites within the region affected by the Fundão Dam disaster. IS, impacted site; PS, preserved site.

| **Voucher number** | **Species** | **Site** |
| --- | --- | --- |
| 204084 | *Bryum atenense* R. S. Williams - Moss  *Schizothrix* Kützing ex Gomont - Cyanobacteria | IS |
| 204085 | *Bryum atenense* R. S. Williams - Moss  *Scytonema* C.Agardh ex É.Bornet & C.Flahault - Cyanobacteria | IS |
| 204086 | *Bryum atenense* R. S. Williams - Moss  *Fissidens zollingeri* Mont. - Moss  *Scytonema* C.Agardh ex É.Bornet & C.Flahault - Cyanobacteria  *Nostoc* Vaucher ex Bornet & Flahault - Cyanobacteria | IS |
| 204087 | *Fissidens zollingeri* Mont. - Moss  *Scytonema* C.Agardh ex É.Bornet & C.Flahault - Cyanobacteria | IS |
| 204088 | *Fissidens zollingeri* Mont. - Moss | IS |
| 204090 | *Fissidens zollingeri* Mont. - Moss  *Bryum orthodontioides* Müll.Hal. - Moss  *Scytonema* C.Agardh ex É.Bornet & C.Flahault - Cyanobacteria | IS |
| 204091 | *Bryum argenteum* Hedw. - Moss  *Bryum atenense* R. S. Williams - Moss  *Scytonema* C.Agardh ex É.Bornet & C.Flahault - Cyanobacteria  *Oscillatoria* Vaucher ex Gomont - Cyanobacteria  *Gloeothece* C. Nägeli - Cyanobacteria | IS |
| 204092 | *Bryum orthodontioides* Müll.Hal. - Moss | IS |
| 204093 | *Philonotis sphaerocarpa* (Hedw.) Brid. - Moss  *Fissidens zollingeri* Mont. - Moss  *Scytonema* C.Agardh ex É.Bornet & C.Flahault - Cyanobacteria  *Gloeothece* C. Nägeli - Cyanobacteria | IS |
| 204094 | *Bryum orthodontioides* Müll.Hal. - Moss  *Fissidens zollingeri* Mont. - Moss  *Gloeothece* C. Nägeli - Cyanobacteria | IS |
| 204095 | *Bryum orthodontioides* Müll.Hal. - Moss | IS |
| 204096 | *Bryum orthodontioides* Müll.Hal. - Moss | IS |
| 204097 | *Bryum orthodontioides* Müll.Hal. - Moss | IS |
| 204098 | *Funaria hygrometrica* Hedw. - Moss  *Scytonema* C.Agardh ex É.Bornet & C.Flahault - Cyanobacteria | IS |
| 204099 | *Fissidens pellucidus* Hornsch. - Moss  *Funaria hygrometrica* Hedw. - Moss | IS |
| 204100 | *Funaria hygrometrica* Hedw. - Moss | IS |
| 204101 | *Hyophila involuta* (Hook.) A. Jaeger - Moss | IS |
| 204102 | *Philonotis sphaerocarpa* (Hedw.) Brid. - Moss | IS |
| 204103 | *Bryum subapiculatum* Hampe - Moss | IS |
| 204104 | *Splachnobryum obtusum* (Brid.) Müll. Hal. – Moss  *Bryum subapiculatum* Hampe - Moss | IS |
| 204105 | *Bryum subapiculatum* Hampe - Moss | IS |
| 204106 | *Bryum subapiculatum* Hampe - Moss | IS |
|  |  |  |

**Supplementary Table A –** Cont.

| **Voucher number** | **Species** | **Site** |
| --- | --- | --- |
| 204107 | *Campylopus heterostachys* (Hampe) A. Jaeger - Moss  *Fissidens zollingeri* Mont. - Moss  *Scytonema* C.Agardh ex É.Bornet & C.Flahault - Cyanobacteria  *Gloeothece* C. Nägeli - Cyanobacteria | PS |
| 204108 | *Bryum orthodontioides* Müll.Hal. - Moss  *Cephaloziella granatensis* (J.B.Jack) Fulford - Liverwort  *Stigonema* C.Agardh ex Bornet & Flahault - Cyanobacteria | PS |
| 204109 | *Dicranella hilariana* (Mont.) Mitt. - Moss  *Scytonema* C.Agardh ex É.Bornet & C.Flahault - Cyanobacteria | PS |
| 204110 | *Campylopus heterostachys* (Hampe) A. Jaeger - Moss  *Fissidens zollingeri* Mont. - Moss  *Bryum orthodontioides* Müll.Hal. - Moss  *Cephaloziella granatensis* (J.B.Jack) Fulford - Liverwort  *Scytonema* C.Agardh ex É.Bornet & C.Flahault - Cyanobacteria  *Gloeothece* C. Nägeli - Cyanobacteria  *Stigonema* C.Agardh ex Bornet & Flahault - Cyanobacteria  *Oscillatoria* Vaucher ex Gomont - Cyanobacteria | PS |
| 204111 | *Fissidens zollingeri* Mont. - Moss | PS |
| 204112 | *Bryum coronatum* Schwägr. - Moss | PS |
| 204113 | *Campylopus heterostachys* (Hampe) A. Jaeger - Moss  *Bryum coronatum* Schwägr. - Moss  *Fissidens zollingeri* Mont. - Moss  *Scytonema* C.Agardh ex É.Bornet & C.Flahault - Cyanobacteria | PS |
| 204114 | *Bryum orthodontioides* Müll.Hal. - Moss  *Bryum coronatum* Schwägr. - Moss  *Microcoleus* Desmazières ex Gomont - Cyanobacteria | PS |
| 204115 | *Tortella tortuosa* (Hedw.) Limpr. – Moss  *Bryum coronatum* Schwägr. - Moss  *Cephaloziella granatensis* (J.B.Jack) Fulford - Liverwort  *Stigonema* C.Agardh ex Bornet & Flahault - Cyanobacteria | PS |
| 204116 | *Tortella tortuosa* (Hedw.) Limpr. – Moss  *Cephaloziella granatensis* (J.B.Jack) Fulford - Liverwort  *Stigonema* C.Agardh ex Bornet & Flahault - Cyanobacteria | PS |
| 204117 | *Vitalia cuspidifera* (Mitt.) P.E.A.S.Câmara, Carv.-Silva & W.R. Buck - Moss | PS |
| 204118 | *Bryum coronatum* Schwägr. - Moss | PS |
| 204119 | *Bryum coronatum* Schwägr. - Moss  *Scytonema* C.Agardh ex É.Bornet & C.Flahault - Cyanobacteria | PS |
| 204120 | *Vitalia cuspidifera* (Mitt.) P.E.A.S.Câmara, Carv.-Silva & W.R. Buck - Moss | PS |
| 204121 | *Tortella tortuosa* (Hedw.) Limpr. - Moss  *Vitalia cuspidifera* (Mitt.) P.E.A.S.Câmara, Carv.-Silva & W.R. Buck - Moss  *Cheilolejeunea discoidea* (Lehm. & Lindenb.) Kachr. & R.M.Schust. - Liverwort  *Cephaloziella granatensis* (J.B.Jack) Fulford - Liverwort  *Scytonema* C.Agardh ex É.Bornet & C.Flahault - Cyanobacteria | PS |
| 204122 | *Vitalia cuspidifera* (Mitt.) P.E.A.S.Câmara, Carv.-Silva & W.R. Buck - Moss  *Chonecolea doellingeri* (Nees) Grolle - Liverwort  *Scytonema* C.Agardh ex É.Bornet & C.Flahault - Cyanobacteria | PS |

**Supplementary Table A –** Cont.

| **Voucher number** | **Species** | **Site** |
| --- | --- | --- |
| 204123 | *Campylopus gardneri* (Müll.Hal.) Mitt. - Moss  *Scytonema* C.Agardh ex É.Bornet & C.Flahault - Cyanobacteria | PS |
| 204124 | *Vitalia cuspidifera* (Mitt.) P.E.A.S.Câmara, Carv.-Silva & W.R. Buck - Moss  *Scytonema* C.Agardh ex É.Bornet & C.Flahault - Cyanobacteria | PS |
| 204125 | *Chryso-hypnum diminutivum* (Hampe) W.R.Buck - Moss | PS |
| 204126 | *Bryum orthodontioides* Müll.Hal. - Moss  *Scytonema* C.Agardh ex É.Bornet & C.Flahault - Cyanobacteria | PS |
| 204127 | *Tortella tortuosa* (Hedw.) Limpr. - Moss  *Vitalia cuspidifera* (Mitt.) P.E.A.S.Câmara, Carv.-Silva & W.R. Buck - Moss | PS |
| 204128 | *Vitalia galipensis* (Müll. Hal.) P.E.A.S.Câmara, Carv.-Silva & W.R. Buck - Moss  *Tortella tortuosa* (Hedw.) Limpr. - Moss  *Brachiolejeunea phyllorhiza* (Nees) Kruijt & Gradst. – Liverworts  *Scytonema* C.Agardh ex É.Bornet & C.Flahault - Cyanobacteria | PS |
| 204129 | *Vitalia cuspidifera* (Mitt.) P.E.A.S.Câmara, Carv.-Silva & W.R. Buck – Moss  *Scytonema* C.Agardh ex É.Bornet & C.Flahault - Cyanobacteria | PS |
| 204130 | *Dicranella hilariana* (Mont.) Mitt. - Moss | PS |
| 204131 | *Bryum coronatum* Schwägr. - Moss  *Fissidens ornatus* Herzog - Moss  *Scytonema* C.Agardh ex É.Bornet & C.Flahault - Cyanobacteria | PS |
| 204132 | *Vitalia cuspidifera* (Mitt.) P.E.A.S.Câmara, Carv.-Silva & W.R. Buck - Moss | PS |
| 204133 | *Vitalia cuspidifera* (Mitt.) P.E.A.S.Câmara, Carv.-Silva & W.R. Buck - Moss  *Tortella tortuosa* (Hedw.) Limpr. - Moss  *Fossombronia porphyrorhiza* (Nees) Prosk. - Liverworts | PS |
| 204134 | *Bryum limbatum* Müll. Hal. - Moss | PS |
| 204135 | *Tortella tortuosa* (Hedw.) Limpr. - Moss  *Fossombronia porphyrorhiza* (Nees) Prosk. - Liverworts  *Cylindrocolea rhizantha* (Mont.) R.M.Schust - Liverworts | PS |
| 204136 | *Vitalia cuspidifera* (Mitt.) P.E.A.S.Câmara, Carv.-Silva & W.R. Buck - Moss  *Tortella tortuosa* (Hedw.) Limpr. - Moss  *Cephaloziella granatensis* (J.B.Jack) Fulford - Liverworts  *Fossombronia porphyrorhiza* (Nees) Prosk. - Liverworts | PS |
| 204137 | *Fossombronia porphyrorhiza* (Nees) Prosk. - Liverworts  *Cephaloziella granatensis* (J.B.Jack) Fulford - Liverworts  *Bryum coronatum* Schwägr. - Moss  *Bryum limbatum* Müll. Hal. - Moss  *Fissidens ornatus* Herzog - Moss | PS |
| 204138 | *Vitalia cuspidifera* (Mitt.) P.E.A.S.Câmara, Carv.-Silva & W.R. Buck - Moss | PS |
| 204139 | *Vitalia cuspidifera* (Mitt.) P.E.A.S.Câmara, Carv.-Silva & W.R. Buck - Moss | PS |
| 204140 | *Octoblepharum albidum* Hedw. - Moss  *Vitalia galipensis* (Müll. Hal.) P.E.A.S.Câmara, Carv.-Silva & W.R. Buck - Moss  *Cheilolejeunea trifaria* var. *clausa* (Nees & Mont.) Gradst. & CJ. Bastos - Liverworts | PS |
| 204141 | *Brittonodoxa subpinnata* (Brid.) W.R. Buck, P.E.A.S.Câmara & Carv.-Silva - Moss  *Octoblepharum albidum* Hedw. - Moss  *Cephaloziella granatensis* (J.B.Jack) Fulford - Liverworts | PS |
