# Supplementary material for: Diversity and life strategies of cyanobacteria and bryophytes within biocrusts in the context of mining tailings disasters in Brazil
Source: Plant Biol (Stuttg). 2025 May 9;27(6):1128–36. doi: 10.1111/plb.70037 (PMC12477303; doi:10.1111/plb.70037)
Supplement: Supplementary file 6 — Table S2. Traits related to bryophyte life strategies, with respective assigned values for categories and description. [file PLB-27-1128-s006.docx]

**Table S2 -** Traits related to bryophyte life strategies, with respective assigned values for categories and description.

| **Traits** | **Categories** | **Description** |
| --- | --- | --- |
| **Spore size** | 1 - Small | Less than 20 µm |
|  | 5 - Median | 21 to 60 µm |
|  | 10 - Large | More than 61 µm |
| **Asexual reproduction** | 1 - Absent | With no production of asexual structures |
|  | 5 - Present | Only one type of asexual structures |
|  | 10 - Diverse | Two or more types of asexual structures |
| **Life form** | 1 - Turfs and cushions | Little resource invested into long-term colony organization |
|  | 5 - Mats and wefts | Moderate resource invested into long-term colony organization |
|  | 10 - Dendroids, fans, pendants | High resource invested into long-term colony organization |
| **Water uptake adaptations** | 1 - Diverse | Two or more types of morphological adaptations |
|  | 5 - Present | Only one type of morphological adaptations |
|  | 10 - Absent | With no morphological adaptations |
| **Sexual system** | 1 - Monoecious | High sexual reproductive success |
|  | 5 - Dioecious | Sexual reproductive success limited by distance |
| **Geographical distribution** | 1 - Wide | Worldwide |
|  | 5 - Intermediate | Only in tropical regions |
|  | 10 - Restricted | Limited to the Neotropics |
