# Supplementary material for: Diversity and life strategies of cyanobacteria and bryophytes within biocrusts in the context of mining tailings disasters in Brazil
Source: Plant Biol (Stuttg). 2025 May 9;27(6):1128–36. doi: 10.1111/plb.70037 (PMC12477303; doi:10.1111/plb.70037)
Supplement: Supplementary file 7 — Table S3. Bryophytes species collected in the mining tailing and preserved site, and their unique values for life strategies based on the sum of trait scores. PS, preserved site; IS, impacted site; BS, both sites; SS, spore size; AR, asexual reproduction; LF, life form; WUA, water uptake adaptations; Sex, sexual system; GD, geographical distribution. *Based on genus information. [file PLB-27-1128-s007.docx]

**Table S3 -** Bryophytes species collected in the mining tailing and preserved site, and their unique values for life strategies based on the sum of trait scores. PS, preserved site; IS, impacted site; BS, both sites; SS, spore size; AR, asexual reproduction; LF, life form; WUA, water uptake adaptations; Sex, sexual system; GD, geographical distribution. *Based on genus information.

| **Specie** | **Collected** | **SS** | **AR** | **LF** | **WUA** | **Sex.** | **GD** | **Total** |
| --- | --- | --- | --- | --- | --- | --- | --- | --- |
| *Brachiolejeunea phyllorhiza* | PS | 10 | 1 | 5 | 1 | 1 | 1 | **19** |
| *Brittonodoxa subpinnata* | PS | 1 | 1 | 5 | 5 | 1 | 5 | **18** |
| *Bryum argenteum* | IS | 1 | 5 | 1 | 1 | 5 | 1 | **14** |
| *Bryum atenense* | IS | 1 | 10 | 1 | 5 | 5 | 10 | **32** |
| *Bryum coronatum* | PS | 1 | 10 | 1 | 5 | 5 | 5 | **27** |
| *Bryum limbatum* | PS | 1 | 1 | 1 | 5 | 5 | 10 | **23** |
| *Bryum orthodontioides* | BS | 1 | 1 | 1 | 5 | 5 | 10 | **23** |
| *Bryum subapiculatum* | IS | 1 | 5 | 1 | 5 | 5 | 10 | **27** |
| *Campylopus gardneri* | PS | 1* | 1 | 1 | 1 | 5 | 10 | **19** |
| *Campylopus heterostachys* | PS | 1 | 1 | 1 | 1 | 5 | 10 | **19** |
| *Cephaloziella granatensis* | PS | 1 | 5 | 5 | 5 | 1 | 10 | **27** |
| *Cheilolejeunea clausa* | PS | 1 | 1 | 5 | 1 | 1 | 5 | **23** |
| *Cheilolejeunea discoidea* | PS | 0 | 5 | 5 | 5 | 1 | 5 | **31** |
| *Chonecolea doellingeri* | PS | 1 | 5 | 5 | 10 | 1 | 5 | **27** |
| *Chryso-hypnum diminutivum* | PS | 0 | 1 | 5 | 10 | 1 | 10 | **28** |
| *Cylindrocolea rhizantha* | PS | 1 | 1 | 5 | 10 | 1 | 10 | **28** |
| *Dicranella hilariana* | PS | 1 | 1 | 1 | 5 | 5 | 5 | **18** |
| *Fissidens ornatus* | PS | 1 | 1 | 10 | 1 | 1 | 10 | **24** |
| *Fissidens pellucidus* | IS | 1 | 1 | 10 | 1 | 1 | 5 | **19** |
| *Fissidens zollingeri* | BS | 1 | 5 | 1 | 1 | 1 | 5 | **14** |
| *Fossombronia porphyrorhiza* | PS | 1 | 5 | 5 | 10 | 1 | 10 | **36** |
| *Funaria hygrometrica* | IS | 1 | 1 | 1 | 5 | 1 | 1 | **10** |
| *Hyophila involuta* | IS | 5 | 5 | 1 | 1 | 5 | 1 | **14** |
| *Octoblepharum albidum* | PS | 1 | 5 | 1 | 5 | 1 | 5 | **22** |
| *Philonotis sphaerocarpa* | IS | 1 | 1 | 1 | 1 | 5 | 5 | **14** |
| *Splachnobryum obtusum* | IS | 5 | 5 | 1 | 5 | 5 | 1 | **18** |
| *Tortella tortuosa* | PS | 1 | 5 | 1 | 1 | 5 | 1 | **14** |
| *Vitalia cuspidifera* | PS | 1 | 1 | 5 | 5 | 1 | 10 | **23** |
| *Vitalia galipensis* | PS | 1 | 1 | 5 | 5 | 1 | 10 | **23** |
