# Supplementary material for: Diversity and life strategies of cyanobacteria and bryophytes within biocrusts in the context of mining tailings disasters in Brazil
Source: Plant Biol (Stuttg). 2025 May 9;27(6):1128–36. doi: 10.1111/plb.70037 (PMC12477303; doi:10.1111/plb.70037)
Supplement: Supplementary file 8 — Table S4. Descriptive results of chemical properties for each sampled area. SD, standard deviation. [file PLB-27-1128-s005.docx]

**Table S4 -** Descriptive results of chemical properties for each sampled area. SD, standard deviation.

| **Soil variables** | **Studied sites** | |
| --- | --- | --- |
|  | **Preserved** | **Impacted** |
|  | Mean ± SD | |
| Phosphorus (P) – mg/dm^3^ | 8.54 ± 4.46 | 1.93 ± 2.90 |
| Potassium (K) – mg/dm^3^ | 132.83 ± 12.74 | 90.22 ± 32.01 |
| Calcium (Ca) – cmolc/dm^3^ | 1.60 ± 0.87 | 0.77 ± 0.46 |
| Magnesium (Mg) – cmolc/dm^3^ | 0.59 ± 0.34 | 0.28 ± 0.08 |
| Sulfur (S) – mg/dm^3^ | 4.80 ± 0.81 | 19.58 ± 6.66 |
| Cupper (Cu) – mg/dm^3^ | 0.40 ± 0.57 | 0.56 ± 0.17 |
| Iron (Fe) – mg/dm^3^ | 90.40 ± 38.45 | 94.56 ± 32.91 |
| Manganese (Mn) – mg/dm^3^ | 34.10 ± 20.13 | 29.72 ± 39.53 |
| Boron (B) – mg/dm^3^ | 0.01 ± 0.00 | 0.01 ± 0.00 |
| Zinc (Zn) – mg/dm^3^ | 2.24 ± 1.01 | 2.82 ± 2.32 |
| Aluminum (Al) – cmolc/dm^3^ | 0.46 ± 0.42 | 0.20 ± 0.16 |
| Sodium (Na) – mg/dm^3^ | 3.20 ± 0.45 | 12.40 ± 23.82 |
| Ph | 4.62 ± 0.29 | 5.04 ± 1.00 |
| Total acidity (H+Al) – cmolc/dm^3^ | 6.34 ± 2.26 | 4.00 ± 1.96 |
| Sum of bases (SB) | 2.53 ± 1.19 | 1.28 ± 0.40 |
| Effective cation exchange capacity (ECEC) | 2.99 ± 0.92 | 1.48 ± 0.32 |
| Cation exchange capacity (CEC) | 8.87 ± 2.04 | 5.28 ± 1.64 |
| Base saturation index (BSI) | 29.76 ± 14.72 | 29.84 ± 24.52 |
| Aluminum saturation index (ASI) | 17.71 ± 17.39 | 14.28 ± 10.73 |
| Organic matter (OM) – dag/Kg | 1.85 ± 0.88 | 1.14 ± 0.38 |
| Remaining phosphorus (PRem) – mg/L | 41.68 ± 7.64 | 22.54 ± 3.06 |
