# Supplementary material for: Diversity and life strategies of cyanobacteria and bryophytes within biocrusts in the context of mining tailings disasters in Brazil
Source: Plant Biol (Stuttg). 2025 May 9;27(6):1128–36. doi: 10.1111/plb.70037 (PMC12477303; doi:10.1111/plb.70037)
Supplement: Supplementary file 9 — Table S5. Site scores on the first and second components of the NMDS based on soil variables. In bold, the most significant variables with P‐values < 0.05 in the NMDS, and with a * those that were used in the MANOVA. [file PLB-27-1128-s008.docx]

**Table S5 -** Site scores on the first and second components of the NMDS based on soil variables. In bold, the most significant variables with *P*-values < 0.05 in the NMDS, and with a * those that were used in the MANOVA.

| **Variable** | **NMDS 1** | **NMDS 2** | ***P*-value** |
| --- | --- | --- | --- |
| Potassium (K) | 0.14255037 | 0.73703324 | 0.065 |
| Phosphorus (P) | 0.66185488 | 0.72052406 | **0.001*** |
| Sodium (Na) | 0.74009543 | -0.49340435 | **0.017** |
| Calcium (Ca) | 0.77395406 | 0.45338149 | **0.004** |
| Magnesium (Mg) | 0.13335888 | 0.61523888 | 0.168 |
| Aluminum (Al) | -0.30408514 | 0.78507222 | **0.013** |
| Zinc (Zn) | 0.06737766 | -0.07235015 | 0.956 |
| Iron (Fe) | 0.09728873 | 0.12256020 | 0.933 |
| Manganese (Mn) | 0.81466475 | -0.24538700 | **0.014** |
| Cupper (Cu) | 0.01920927 | -0.30835755 | 0.694 |
| Sulfur (S) | -0.44519550 | -0.84596821 | **0.001*** |
| Boron (B) | 0.000000000 | 0.000000000 | 1.000 |
| pH | 0.61824811 | -0.74296597 | **0.001*** |
| Total acidity (H+Al) | -0.42700999 | 0.83193035 | **0.004*** |
| Sum of bases (SB) | 0.61174254 | 0.61062507 | **0.009** |
| Effective cation exchange capacity (ECEC) | 0.42675802 | 0.83518983 | **0.001*** |
| Cation exchange capacity (CEC) | -0.17407152 | 0.88604726 | **0.005*** |
| Base saturation index (BSI) | 0.88027159 | -0.39882781 | **0.001*** |
| Aluminum saturation index (ASI) | -0.73436914 | 0.62044366 | **0.001*** |
| Organic matter (OM) | 0.01712764 | 0.57856541 | 0.226 |
| Remaining phosphorus (PRem) | 0.33490010 | 0.81880372 | **0.009** |
