# Supplementary material for: Diversity and life strategies of cyanobacteria and bryophytes within biocrusts in the context of mining tailings disasters in Brazil
Source: Plant Biol (Stuttg). 2025 May 9;27(6):1128–36. doi: 10.1111/plb.70037 (PMC12477303; doi:10.1111/plb.70037)
Supplement: Supplementary file 10 — Table S6. Summary of MANOVA for the soil variables across the study sites. Significant P‐values are in bold; df, degrees of freedom. [file PLB-27-1128-s002.docx]

**Table S6 -** Summary of MANOVA for the soil variables across the study sites. Significant *P*-values are in bold; df = degrees of freedom.

| **MANOVA** | **Pillai’s trace** | **F** | **P** |
| --- | --- | --- | --- |
| **Studied sites** | 0.99999 | 20597 | **0.00539** |
| **Response** | **df** | **F** | **P** |
| Phosphorus (P) | 1 | 12.536 | **0.0076** |
| Sulfur (S) | 1 | 41.585 | **0.0002** |
| pH | 1 | 0.792 | 0.3995 |
| Total acidity (H+Al) | 1 | 2.218 | 0.1747 |
| Effective cation exchange capacity (ECEC) | 1 | 18.305 | **0.0027** |
| Cation exchange capacity (CEC) | 1 | 7.378 | **0.0264** |
| Base saturation index (BSI) | 1 | 0.061 | 0.8109 |
| Aluminum saturation index (ASI) | 1 | 0.076 | 0.7898 |
